# The crystal structures of two isomers of 5-(phenyl­iso­thia­zol­yl)-1,3,4-oxa­thia­zol-2-one

**DOI:** 10.1107/S2056989017015067

**Published:** 2017-10-20

**Authors:** Shuguang Zhu, Melbourne J. Schriver, Arthur D. Hendsbee, Jason D. Masuda

**Affiliations:** a Teva Pharmaceuticals, 3333 N Torrey Pines Ct, Suite 400, La Jolla, CA 92130; b Department of Chemistry, Crandall University, PO Box 6004, Moncton, New Brunswick, E1C 9L7, Canada; c The Atlantic Centre for Green Chemistry and the Department of Chemistry, Saint Mary’s University, Halifax, Nova Scotia, B3H 3C3, Canada

**Keywords:** crystal structure, iso­thiazo­yl, oxa­thia­zolone, conjugation, nitrile sulfide, π-stacking

## Abstract

The syntheses and crystal structures of two isomers of phenyl iso­thia­zolyl oxa­thia­zolone, C_11_H_6_N_2_O_2_S_2_, are described [systematic names: 5-(3-phenyl­iso­thia­zol-5-yl)-1,3,4-oxa­thia­zol-2-one, (I), and 5-(3-phenyl­iso­thia­zol-4-yl)-1,3,4-oxa­thia­zol-2-one, (II)]. There are two almost planar (r.m.s. deviations = 0.032 and 0.063 Å) mol­ecules of isomer (I) in the asymmetric unit, which form centrosymmetric tetra­mers linked by strong S⋯N [3.072 (2) Å] and S⋯O contacts [3.089 (1) Å]. The tetra­mers are π-stacked parallel to the *a*-axis direction. The single mol­ecule in the asymmetric unit of isomer (II) is twisted into a non-planar conformation by steric repulsion [dihedral angles between the central iso­thia­zolyl ring and the pendant oxa­thia­zolone and phenyl rings are 13.27 (6) and 61.18 (7)°, respectively], which disrupts the π-conjugation between the heteroaromatic iso­thia­zoloyl ring and the non-aromatic oxa­thia­zolone heterocycle. In the crystal of isomer (II), the strong S⋯O [3.020 (1) Å] and S⋯C contacts [3.299 (2) Å] and the non-planar structure of the mol­ecule lead to a form of π-stacking not observed in isomer (I) or other oxa­thia­zolone derivatives.

## Abstract 2

The 3,5-isomer of the title compound contains two almost planar mol­ecules in the asymmetric unit, whereas the 3,4-isomer contains a single substanti­ally twisted mol­ecule. Both crystal structures feature short S⋯N and S⋯O inter­actions.

## Chemical context   

Compounds containing the iso­thia­zolyl moiety are well known in organic and pharmacological research, with extensive reviews on the synthesis and chemistry of the ring (Abdel-Sattar & Elgazwy, 2003 ▸) and the medicinal and industrial uses of compounds containing the iso­thia­zolyl heterocycle (Kaberdin & Potkin 2002 ▸). The solid-state structural features of iso­thia­zole derivatives have been reviewed (Abdel-Sattar & Elgazwy, 2003 ▸). In general, the iso­thia­zolyl ring is recognised as a heteroaromatic ring with extensive π-delocalization (incorporating the empty sulfur 3*d*-orbitals) within the ring leading to almost planar heterocycles.

Derivatives of the oxa­thia­zolone heterocycle have been known since their first preparation fifty years ago (Muhlbauer & Weiss, 1967 ▸). The facile synthesis of the heterocycle from commercially available amides reacting with chloro­carbonyl sulfenyl chloride under a range of conditions has resulted in the publication of significant libraries of substituted oxa­thia­zolone compounds (Senning & Rasmussen, 1973 ▸; Howe *et al.*, 1978 ▸; Lin *et al.*, 2009 ▸; Fordyce *et al.*, 2010 ▸; Russo *et al.*, 2015 ▸) leading to hundreds of known oxa­thia­zolone derivatives. The predominant chemistry of the heterocycle has been the thermal cyclo­reversion to the short lived nitrile sulfide [*R*—C≡N^(+)^—S^(−)^] , a propargyl allenyl 1,3-dipole, which can be trapped by electron-deficient π bonds in reasonable yield to give families of new heterocycles (Paton, 1989 ▸), including iso­thia­zole derivatives. As a result of the electronic properties of the short-lived nitrile sulfide inter­mediates, optimal conditions for cyclization require trapping reactions with electron-deficient dipolariphiles. Industrially, various derivatives of the oxa­thia­zolone heterocycle have been reported as potential fungicides (Klaus *et al.*, 1965 ▸), pesticides (Hölzl, 2004 ▸) and as polymer additives (Crosby 1978 ▸). More recently, the medicinal properties of the oxa­thia­zolone heterocycle have been explored as selective inhibitors for tuberculosis (Lin *et al.*, 2009 ▸), inflammatory diseases (Fan *et al.*, 2014 ▸) and as proteasome inhibitors (Russo *et al.*, 2015 ▸).

In previous structural studies on oxa­thia­zolone compounds, the non-aromatic heterocyclic rings were found to be planar with largely localized C=N and C=O double bonds. The extent of π-delocalization within the oxa­thia­zolone ring and to the substituent group and the effect on the structure and chemical properties have been discussed spectroscopically (Markgraf *et al.*, 2007 ▸) and structurally (Krayushkin *et al.*, 2010*a* ▸,*b* ▸). Our inter­est in this system was prompted by the possibility that catenated systems of iso­thia­zolone heterocycles may have useful electronic properties as the number of π systems is increased.
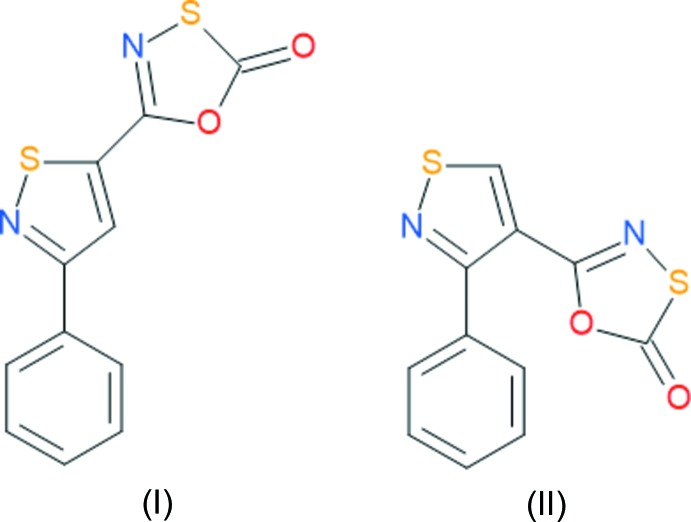


## Structural commentary   

**Figure 1:**
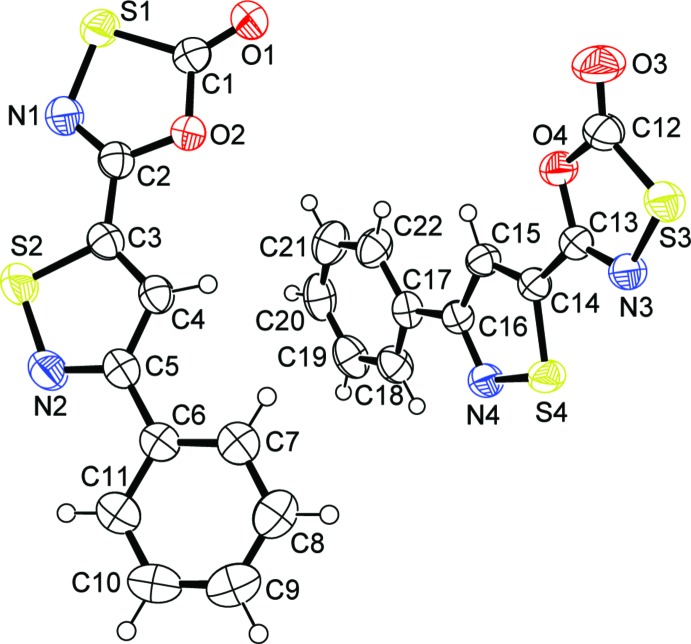
The mol­ecular structure of (I), showing 50% probability displacement ellipsoids.

**Figure 2:**
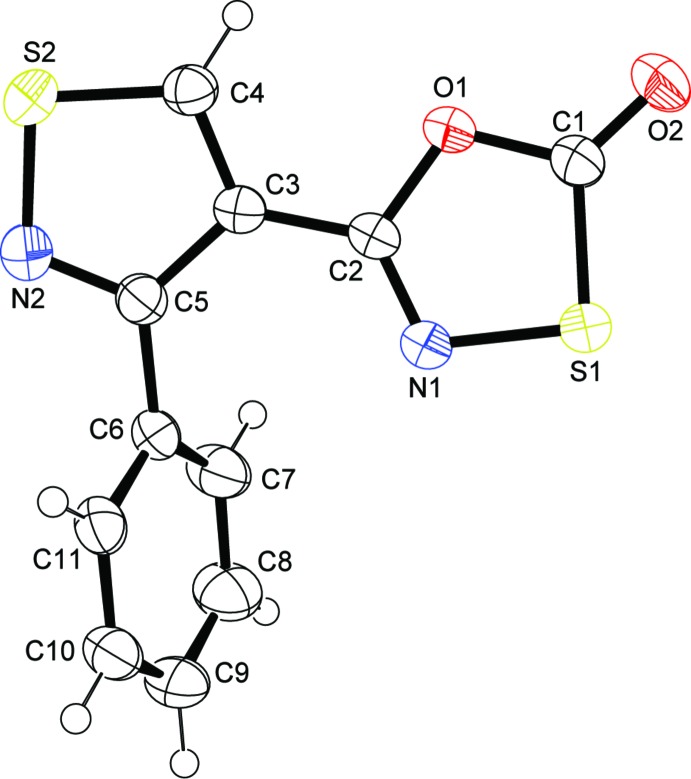
The mol­ecular structure of (II), showing 50% probability displacement ellipsoids.

There are two independent mol­ecules in the asymmetric unit of (I) (Fig. 1 ▸). In general, the two mol­ecules are not significantly different with the exception of the C—S bonds in the oxa­thia­zolone rings. In one of the mol­ecules, the C1—S1 distance [1.762 (2) Å] is longer than the same bond in the second mol­ecule, C12—S3 [1.746 (2) Å]. The difference may arise from the nature of the inter­molecular contacts to the sulfur atoms, with a strong pair of co-planar S⋯N contacts [3.086 (2) Å] in the first mol­ecule but only one S⋯N contact [3.072 (2) Å] in the second mol­ecule (which is also twisted out of the plane of the mol­ecule). These differences are due to the position of the independent mol­ecules in the tetra­mer that will be described below. For the purposes of further structural analysis, we will restrict our discussion to the first mol­ecule in the asymmetric unit. The asymmetric unit of (II) is shown in Fig. 2 ▸.

The bond distances and angles within the terminal phenyl rings in compounds (I) and (II) are not significantly different from the those reported for related compounds (Schriver & Zaworotko, 1995 ▸; Krayushkin *et al.*, 2010*a* ▸,*b* ▸). The sum of the endocyclic bond angles in the iso­thia­zole moieties for both (I) and (II) (540.0°) is consistent with planar (ideal sum = 540°) π-delocalized five-membered rings, as expected. The bond lengths of the endocyclic bonds in the iso­thia­zolyl moieties in (I) and (II) are not significantly (δ > 3σ) different from the statistical averages from previous structural studies (Bridson *et al.*, 1994 ▸, 1995 ▸). While the C=N bonds in the iso­thia­zolyl rings of (I) [1.327 (3) Å] and (II) [1.321 (2) Å] and the C=C bonds in (I) [1.361 (3) Å] and (II) [1.374 (2) Å] are mostly longer than the statistical averages for C=N [1.308 ± 0.016 Å] and C=C bonds [1.369 ± 0.002 Å], the differences are not sufficient to warrant an assessment of their cause or their effect on the structure.

The bond distances and angles within the oxa­thia­zolone rings in compounds (I) and (II) are not significantly different (δ ≥ 3σ) from the statistical averages for published crystal structures (Schriver & Zaworotko, 1995 ▸; Bridson *et al.* 1994 ▸, 1995 ▸; Vorontsova *et al.*, 1996 ▸; McMillan *et al.*, 2006 ▸; Krayushkin *et al.*, 2010*a* ▸,*b* ▸; Nason *et al.*, 2017 ▸). The sum of the endocyclic bond angles in the oxa­thia­zolone rings for both (I) and (II) (540.0°) is consistent with planar rings (ideal sum = 540°). The S—N bonds in the oxa­thia­zolone rings of (I) [1.685 (2) Å] and (II) [1.682 (1) Å], the C_sub_—O bonds in (I) [1.364 (2) Å] and (II) [1.375 (1) Å] and the inter-ring C*sp* ^2^—C*sp* ^2^ bonds in (I) [1.449 (3) Å] and (II) [1.451 (2) Å] are all consistently shorter than the statistical averages for S—N [1.696 ± 0.022 Å], C_sub_—O [1.392 ± 0.030 Å] and C=C bonds [1.461 ± 0.025 Å]. These differences, however, are not sufficient to warrant an assessment of their cause or their effect on the structure.

The three rings in the mol­ecules of (I) are nearly co-planar, with the dihedral angles between central iso­thia­zolyl ring and the pendant oxa­thia­zolone and phenyl rings being 3.06 (11) and 1.10 (12)°, respectively, for the S1 mol­ecule and 2.62 (9) and 6.84 (10)°, respectively, for the S3 mol­ecule. Overall r.m.s. deviations for the S1 and S3 mol­ecules are 0.032 and 0.063 Å, respectively. In contrast to the near planarity of both asymmetric mol­ecules of (I), the single mol­ecule of (II) features significant twists between the central iso­thia­zolyl ring and the pendant oxa­thia­zolone and phenyl rings [dihedral angles of 13.27 (6) and 61.18 (7)°, respectively], which may be ascribed to steric crowding. It has been argued, based on spectroscopic and structural evidence, that π-delocalization extends between the rings of oxa­thia­zolone heterocycles attached to aromatic rings, resulting in observable differences (Schriver & Zaworotko, 1995 ▸; Krayushkin *et al.*, 2010*a* ▸,*b* ▸; Markgraf *et al.*, 2007 ▸). In this work it can be seen that nearly identical mol­ecules result, even when torsion angles are present that would effectively disrupt any π conjugation between the rings, suggesting that the presence or absence of inter-ring π delocalization does not have a significant effect on the structure of the mol­ecules.

## Supra­molecular features   

**Figure 3:**
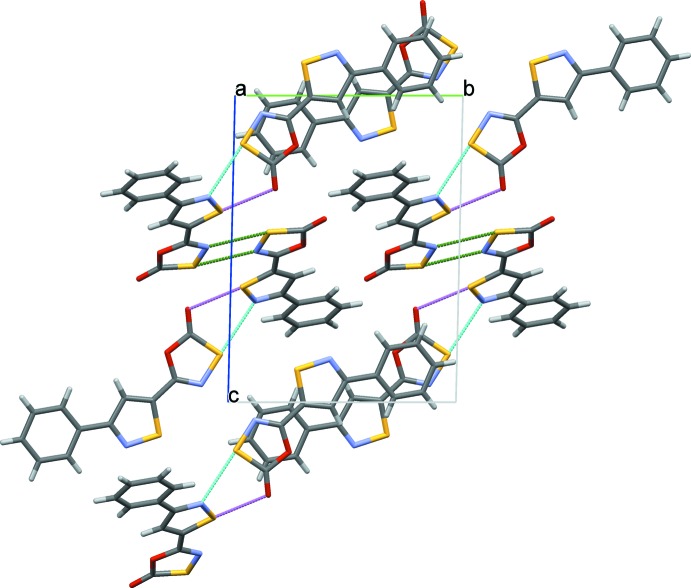
A packing diagram of (I) showing π–π stacking parallel to the *a*-axis direction (top). Co-planar paired head-to-head mol­ecules [green lines, S⋯N distance of 3.086 (2) Å] and paired mol­ecules separated by out-of-plane contacts [blue lines, S⋯N distance of 3.072 (2) Å], violet lines S⋯O distance of 3.089 (1) Å].

In all previous reports on the solid-state structures of compounds containing the oxa­thia­zolone heterocycle, the inter­molecular inter­actions have been ignored or described as insignificant, with the exception of the recent observation of π-stacking in the styryl derivative (Nason *et al.*, 2017 ▸). The strongest inter­molecular contacts in (I) are S3⋯N3 [3.086 (2) Å], S1⋯N4 [3.072 (2) Å] and S4⋯O1 [3.089 (1) Å] (Fig. 3 ▸). The S3⋯N3 contacts assist in the formation of a co-planar pair of identical mol­ecules within the asymmetric unit. The other mol­ecules in the asymmetric unit are connected *via* the S1⋯N4 [3.072 (2) Å] and S4⋯O1 [3.089 (1) Å] contacts. Taken together, the contacts between two pairs of identical mol­ecules in the asymmetric unit form a centrosymmetric tetra­mer that in turn form π-stacks parallel to the *a* axis. The inter­molecular contacts between sulfur and nitro­gen and oxygen have been observed in another oxa­thia­zolone ring that also resulted in π-stacking of the planar mol­ecules (Nason *et al.*, 2017 ▸).

**Figure 4:**
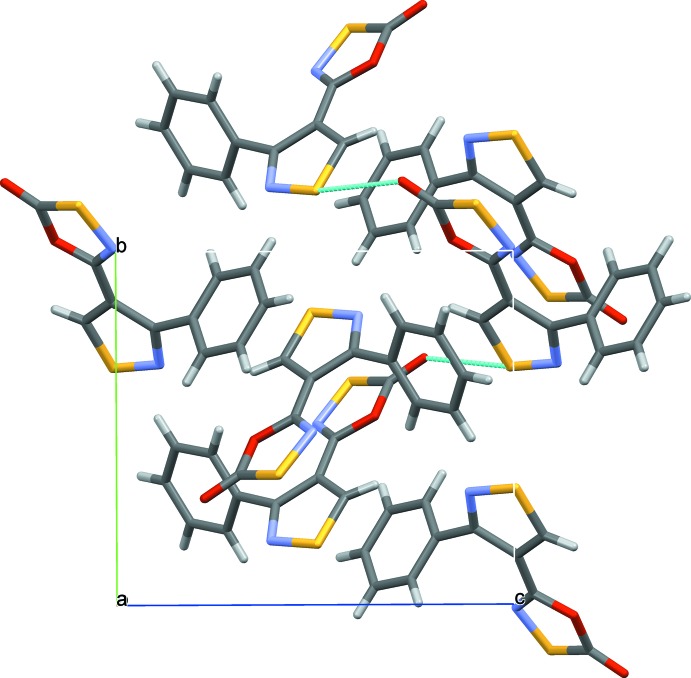
A packing diagram of (II) within the unit cell showing mol­ecular pairs linked by S⋯O contacts of 3.020 (1) Å.

The strongest inter­molecular contacts in (II) are S2⋯O2 [3.020 (1) Å], S1⋯C10 [3.299 (2) Å] and C4⋯O2 [3.100 (2) Å] (Fig. 4 ▸). The C4⋯O2 contact, while significantly shorter than the sum of van der Waals radii for the atoms, is to some extent, the result of the adjacent stronger S2⋯O2 contact. The geometry of the mol­ecule (II) reduces the opportunity for the formation of π-stacks but it is observed that the centroid of the terminal phenyl ring is 3.632 (2) Å above and parallel to the nearly planar portion of an adjacent mol­ecule formed by the two heterocyclic rings (Fig. 4 ▸).

### Database survey   

A search of the Cambridge Structural Database (Version 5.38; Groom *et al.*, 2016 ▸) revealed that eleven crystal structures of oxa­thia­zolone derivatives in peer-reviewed journals have been reported previously (Bridson *et al.*, 1994 ▸, 1995 ▸; Schriver & Zaworotko, 1995 ▸; Vorontsova *et al.*, 1996 ▸; McMillan *et al.*, 2006 ▸; Krayushkin *et al.*, 2010*a* ▸,*b* ▸; Nason *et al.*, 2017 ▸), which have been partially reviewed (McMillan *et al.*, 2006 ▸ and Krayushkin *et al.*, 2010*a* ▸,*b* ▸). An additional five X-ray oxa­thia­zolone crystal structures have been reported in theses (Demas, 1982 ▸; Zhu, 1997 ▸). There are also two published gas-phase electron-diffraction structures of oxa­thia­zolone derivatives (Bak *et al.*, 1978 ▸, 1982 ▸). The structures fall into two groups: those that feature a C*sp* ^2^—C*sp* ^3^ bond between the heterocycle and the saturated organic substituent and those that feature a C*sp* ^2^—C*sp* ^2^ bond between the heterocycle and the unsaturated organic substituent (either a phenyl group, heterocyclic ring or alkenyl moiety).

## Synthesis and crystallization   

**Figure 5:**
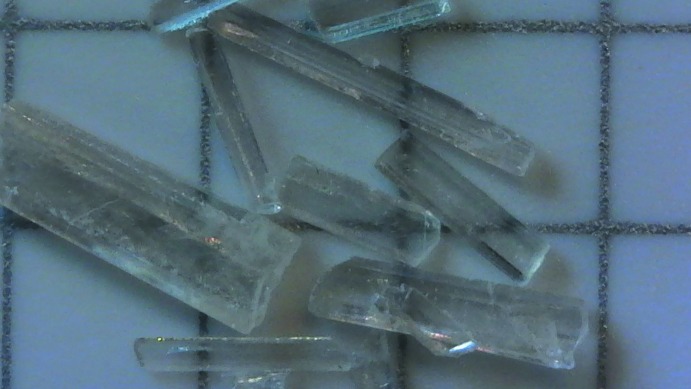
A photograph of crystals of (I) (5 × 5 mm background grid).

**Compound (I)** was prepared following a local variation of literature methods (Howe *et al.*, 1978 ▸). 3-Phenyl­iso­thia­zole-4-carbonamide (Zhu, 1997 ▸) (2.90 g, 14.2 mmol) was placed in 50 ml of toluene under nitro­gen and chloro­carbonyl sulfenyl chloride (4.20 g, 32.0 mmol, approximately 2 × molar excess) was added dropwise to the stirred solution. The resulting mixture was heated (363–373 K) under nitro­gen for 1.5 h and allowed to evaporate to a solid residue. The evaporate was recrystallized from toluene solution to give colourless needle-shaped crystals (Fig. 5 ▸) (3.20 g, 12.2 mmol, 86%). Elemental analysis: calculated % (Found %): 50.35 (50.2); H 2.3 (2.4); N 10.7 (10.7). IR (KBr): 3100 (*w*), 1812 (*w*), 1749 (*s*), 1735 (*s*), 1598 (*s*), 1182 (*m*), 1088 (*m*), 1014 (*w*), 959 (*s*), 884 (*ms*). 834 (*ms*), 765 (*s*), 734 (*s*), 692 (*ms*). ^1^H NMR (400 MHz, CDCl_3_, δ p.p.m.): 9.28 (5, 1H), 7.61 (*m*, 2H), 7.46 (*m*, 3H). ^13^C NMR (100 MHz, CDCl_3_, δ p.p.m.): 172.7,167.0, 154.1, 152.1, 134.0, 129.6, 129.0, 128.2, 123.3. MS (EI): C_11_H_6_N_2_O_2_S_2_ requires (*M* ^+^), 262.301, found *m*/*e* (%, assign.): 262 (22, *M*+), 218 (2, *M-*-CO_2_), 188 (78, *M*–CONS), 186 (100, C_6_H_5_[CCCNS)CN), 160 (1 3, *M*–COCONS), 135 (26, C_6_H_5_CNS), 103 (13, C_6_H_5_CN), 77 (29, C_6_H_5_). UV–visible spectroscopy (hexa­ne) λ_xax_ (log ∊) : 275–230 nm (4.11), 197 nm (4.72).

**Figure 6:**
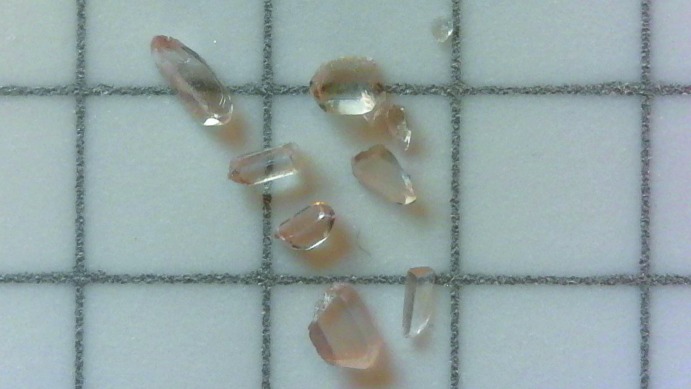
A photograph of crystals of (II) (5 × 5 mm background grid).

**Compound (II)** prepared following a local variation of literature methods (Howe *et al.*, 1978 ▸). 3-Phenyl­iso­thia­zole-5-carbonamide (Zhu, 1997 ▸) (4.08 g, 20.0 mmol) was placed in 50 ml of toluene under nitro­gen and chloro­carbonyl sulfenyl chloride (6.50 g, 50.0 mmol, approximately 2.5 × molar excess) was added dropwise to the stirred solution. The resulting mixture was heated (363–373 K) under nitro­gen for 8.5 h and allowed to evaporate to a solid residue (6.093 g). The evaporate was recrystallized from toluene solution to give colourless block-shaped crystals (Fig. 6 ▸) (4.20 g, 20.6 mmol, 83%), Elemental analysis: calculated % (found%) 50.35 (50.0); H 2.3 (2.35); N 10.7 (10.5). IR (KBr): 3097 (*w*), 3066 (*w*), 3032 (*w*), 1813 (*ms*), 1759 (*s*), 1738 (*s*), 1600 (*ms*), 1590 (*ms*), 1517 (*s*), 1496 (*s*), 1055 (*ms*), 973 (*s*), 902 (*s*), 776 (*s*), 695 (*s*) cm^−1. 1^H NMR (400 MHz, CDCl_3_, δ p.p.m.): 7.425–7.487 (*m*, 3H), 7.906–7.937 (*m*, 2H), 7.976 (5, 1H). ^13^C NMR (100MHz, CDCl_3_, δ p.p.m.): 171.5, 167.9, 150.7, 150.5, 133.4, 129.9, 128.9, 126.8, 122.5. MS (EI): C_11_H_6_N_2_O_2_S_2_ requires (*M* ^+^), 262.301, found *m*/*e* (%, assign.): 262 (52, *M*+), 218 (3, M-CO_2_), 188 (100, *M*–CONS), 160 (9, *M*–COCONS), 135 (2, *M*–HC–C­COCONS). UV–visible spectroscopy (hexa­ne) λ_xax_ (log ∊) : 283 nm (4.25), 248 nm (4.36), 203 nm (94.49).

## Refinement   

Crystal data, data collection and structure refinement details are summarized in Table 1 ▸. H atoms were positioned geometrically (C—H = 0.93 Å) and refined as riding with *U*_iso_(H) = 1.2*U*_eq_(C).

## Supplementary Material

Crystal structure: contains datablock(s) I, II, ms003_0m. DOI: 10.1107/S2056989017015067/hb7705sup1.cif

Structure factors: contains datablock(s) I. DOI: 10.1107/S2056989017015067/hb7705Isup2.hkl

Structure factors: contains datablock(s) II. DOI: 10.1107/S2056989017015067/hb7705IIsup3.hkl

Supporting information file. DOI: 10.1107/S2056989017015067/hb7705Isup4.cml

Supporting information file. DOI: 10.1107/S2056989017015067/hb7705IIsup5.cml

CCDC references: 1580338, 1580337

Additional supporting information:  crystallographic information; 3D view; checkCIF report

## Figures and Tables

**Table 1:** Experimental details

	(I)	(II)
Crystal data		
Chemical formula	C_11_H_6_N_2_O_2_S_2_	C_11_H_6_N_2_O_2_S_2_
*M*_r_	262.30	262.30
Crystal system, space group	Triclinic, *P* 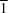	Monoclinic, *P*2_1_/*c*
Temperature (K)	296	296
*a*, *b*, *c* (Å)	7.2739 (7), 11.2713 (11), 14.6909 (15)	9.7202 (6), 9.9723 (6), 11.2165 (7)
α, β, γ (°)	87.562 (1), 78.341 (1), 71.624 (1)	90, 90.399 (1), 90
*V* (Å^3^)	1119.16 (19)	1087.22 (12)
*Z*	4	4
Radiation type	Mo *K*α	Mo *K*α
μ (mm^−1^)	0.46	0.48
Crystal size (mm)	0.49 × 0.25 × 0.14	0.48 × 0.43 × 0.37

Data collection		
Diffractometer	Bruker APEXII CCD	Bruker APEXII CCD
Absorption correction	Multi-scan (*SADABS*; Bruker, 2008 ▸)	Multi-scan (*SADABS*; Bruker, 2008 ▸)
*T*_min_, *T*_max_	0.804, 0.936	0.719, 0.837
No. of measured, independent and observed [*I* > 2σ(*I*)] reflections	7476, 3862, 3485	8041, 2362, 2228
*R*_int_	0.015	0.017
(sin θ/λ)_max_ (Å^−1^)	0.595	0.639

Refinement		
*R*[*F* ^2^ > 2σ(*F* ^2^)], *wR*(*F* ^2^), *S*	0.031, 0.106, 1.04	0.030, 0.083, 1.02
No. of reflections	3862	2362
No. of parameters	308	155
H-atom treatment	H-atom parameters constrained	H-atom parameters constrained
Δρ_max_, Δρ_min_ (e Å^−3^)	0.33, −0.23	0.37, −0.28

Computer programs: *APEX2* and *SAINT* (Bruker, 2008 ▸), *SHELXS97* (Sheldrick, 2008 ▸) and *SHELXL2014* (Sheldrick, 2015 ▸).

## supplementary crystallographic information

### 5-(3-Phenylisothiazol-5-yl)-1,3,4-oxathiazol-2-one (I) Crystal data

**Table:**

C_11_H_6_N_2_O_2_S_2_	*Z* = 4
*M**_r_* = 262.30	*F*(000) = 536
Triclinic, *P*1	*D*_x_ = 1.557 Mg m^−^^3^
*a* = 7.2739 (7) Å	Mo *K*α radiation, λ = 0.71073 Å
*b* = 11.2713 (11) Å	Cell parameters from 5699 reflections
*c* = 14.6909 (15) Å	θ = 2.4–28.6°
α = 87.562 (1)°	µ = 0.46 mm^−^^1^
β = 78.341 (1)°	*T* = 296 K
γ = 71.624 (1)°	Needle, colourless
*V* = 1119.16 (19) Å^3^	0.49 × 0.25 × 0.14 mm

### 5-(3-Phenylisothiazol-5-yl)-1,3,4-oxathiazol-2-one (I) Data collection

**Table:**

Bruker APEXII CCD diffractometer	3485 reflections with *I* > 2σ(*I*)
Radiation source: fine-focus sealed tube	*R*_int_ = 0.015
φ and ω scans	θ_max_ = 25.0°, θ_min_ = 1.9°
Absorption correction: multi-scan (SADABS; Bruker, 2008)	*h* = −5→8
*T*_min_ = 0.804, *T*_max_ = 0.936	*k* = −13→13
7476 measured reflections	*l* = −17→17
3862 independent reflections	

### 5-(3-Phenylisothiazol-5-yl)-1,3,4-oxathiazol-2-one (I) Refinement

**Table:**

Refinement on *F*^2^	Hydrogen site location: inferred from neighbouring sites
Least-squares matrix: full	H-atom parameters constrained
*R*[*F*^2^ > 2σ(*F*^2^)] = 0.031	*w* = 1/[σ^2^(*F*_o_^2^) + (0.0697*P*)^2^ + 0.2729*P*] where *P* = (*F*_o_^2^ + 2*F*_c_^2^)/3
*wR*(*F*^2^) = 0.106	(Δ/σ)_max_ < 0.001
*S* = 1.04	Δρ_max_ = 0.33 e Å^−^^3^
3862 reflections	Δρ_min_ = −0.23 e Å^−^^3^
308 parameters	Extinction correction: SHELXL2014 (Sheldrick, 2015), Fc^*^=kFc[1+0.001xFc^2^λ^3^/sin(2θ)]^-1/4^
0 restraints	Extinction coefficient: 0.031 (3)
Primary atom site location: structure-invariant direct methods	

### 5-(3-Phenylisothiazol-5-yl)-1,3,4-oxathiazol-2-one (I) Special details

**Table:**

Geometry. All esds (except the esd in the dihedral angle between two l.s. planes) are estimated using the full covariance matrix. The cell esds are taken into account individually in the estimation of esds in distances, angles and torsion angles; correlations between esds in cell parameters are only used when they are defined by crystal symmetry. An approximate (isotropic) treatment of cell esds is used for estimating esds involving l.s. planes.

### 5-(3-Phenylisothiazol-5-yl)-1,3,4-oxathiazol-2-one (I) Fractional atomic coordinates and isotropic or equivalent isotropic displacement parameters (Å^2^)

**Table:**

	*x*	*y*	*z*	*U*_iso_*/*U*_eq_	
S1	0.85294 (8)	0.03895 (4)	0.15683 (4)	0.05223 (17)	
O1	0.8238 (2)	0.18576 (13)	0.30252 (9)	0.0593 (4)	
N1	0.8263 (3)	0.12000 (15)	0.05875 (12)	0.0510 (4)	
C1	0.8229 (3)	0.17293 (17)	0.22295 (13)	0.0446 (4)	
S2	0.77918 (10)	0.29901 (5)	−0.10629 (4)	0.05823 (18)	
O2	0.7971 (2)	0.27252 (11)	0.16249 (8)	0.0443 (3)	
N2	0.7537 (3)	0.44255 (17)	−0.14107 (11)	0.0541 (4)	
C2	0.8016 (3)	0.23440 (17)	0.07498 (12)	0.0415 (4)	
S3	0.59888 (8)	0.83750 (5)	0.55508 (4)	0.05152 (17)	
O3	0.6679 (3)	0.59670 (16)	0.59752 (14)	0.0797 (5)	
N3	0.4193 (2)	0.89051 (14)	0.49359 (11)	0.0454 (4)	
C3	0.7806 (3)	0.33109 (18)	0.00604 (12)	0.0415 (4)	
S4	0.09624 (8)	0.94818 (4)	0.37616 (4)	0.05119 (17)	
O4	0.4345 (2)	0.68529 (12)	0.51338 (9)	0.0472 (3)	
N4	−0.0487 (3)	0.89146 (15)	0.33086 (11)	0.0479 (4)	
C4	0.7628 (3)	0.45417 (17)	0.01441 (12)	0.0419 (4)	
H4B	0.7607	0.4931	0.0693	0.050*	
C5	0.7479 (3)	0.51552 (18)	−0.07130 (12)	0.0410 (4)	
C6	0.7266 (3)	0.64887 (18)	−0.08776 (13)	0.0425 (4)	
C7	0.7228 (3)	0.72818 (19)	−0.01704 (14)	0.0503 (5)	
H7A	0.7348	0.6969	0.0418	0.060*	
C8	0.7015 (3)	0.8528 (2)	−0.03326 (18)	0.0604 (6)	
H8A	0.6980	0.9050	0.0149	0.073*	
C9	0.6854 (3)	0.9007 (2)	−0.12035 (19)	0.0652 (6)	
H9A	0.6719	0.9847	−0.1312	0.078*	
C10	0.6895 (4)	0.8231 (2)	−0.19106 (17)	0.0658 (6)	
H10A	0.6784	0.8551	−0.2498	0.079*	
C11	0.7101 (3)	0.6982 (2)	−0.17573 (15)	0.0553 (5)	
H11A	0.7129	0.6467	−0.2242	0.066*	
C12	0.5796 (3)	0.68671 (19)	0.56116 (15)	0.0526 (5)	
C13	0.3557 (3)	0.80060 (16)	0.47969 (12)	0.0396 (4)	
C14	0.2024 (3)	0.81178 (16)	0.42756 (12)	0.0399 (4)	
C15	0.1224 (3)	0.72317 (17)	0.40912 (12)	0.0411 (4)	
H15A	0.1576	0.6415	0.4299	0.049*	
C16	−0.0219 (3)	0.77277 (16)	0.35387 (12)	0.0400 (4)	
C17	−0.1434 (3)	0.70474 (18)	0.32326 (12)	0.0430 (4)	
C18	−0.2967 (3)	0.7675 (2)	0.27896 (14)	0.0523 (5)	
H18A	−0.3229	0.8525	0.2681	0.063*	
C19	−0.4109 (3)	0.7038 (2)	0.25083 (15)	0.0595 (6)	
H19A	−0.5127	0.7462	0.2207	0.071*	
C20	−0.3750 (3)	0.5778 (2)	0.26715 (16)	0.0622 (6)	
H20A	−0.4528	0.5355	0.2485	0.075*	
C21	−0.2239 (4)	0.5153 (2)	0.31101 (18)	0.0665 (6)	
H21A	−0.1988	0.4303	0.3219	0.080*	
C22	−0.1083 (3)	0.5782 (2)	0.33915 (16)	0.0551 (5)	
H22A	−0.0063	0.5351	0.3690	0.066*	

### 5-(3-Phenylisothiazol-5-yl)-1,3,4-oxathiazol-2-one (I) Atomic displacement parameters (Å^2^)

**Table:**

	*U*^11^	*U*^22^	*U*^33^	*U*^12^	*U*^13^	*U*^23^
S1	0.0666 (4)	0.0408 (3)	0.0557 (3)	−0.0208 (2)	−0.0206 (2)	0.0026 (2)
O1	0.0852 (11)	0.0496 (8)	0.0412 (8)	−0.0173 (7)	−0.0144 (7)	0.0018 (6)
N1	0.0606 (11)	0.0476 (9)	0.0501 (9)	−0.0188 (8)	−0.0199 (8)	−0.0004 (7)
C1	0.0459 (10)	0.0403 (9)	0.0471 (11)	−0.0130 (8)	−0.0093 (8)	0.0029 (8)
S2	0.0818 (4)	0.0563 (3)	0.0435 (3)	−0.0271 (3)	−0.0187 (3)	−0.0022 (2)
O2	0.0543 (8)	0.0386 (7)	0.0404 (7)	−0.0131 (6)	−0.0127 (6)	0.0008 (5)
N2	0.0673 (11)	0.0586 (10)	0.0407 (9)	−0.0234 (9)	−0.0148 (8)	0.0033 (7)
C2	0.0387 (9)	0.0452 (10)	0.0420 (9)	−0.0133 (8)	−0.0108 (7)	−0.0007 (7)
S3	0.0545 (3)	0.0546 (3)	0.0554 (3)	−0.0224 (2)	−0.0255 (2)	0.0045 (2)
O3	0.0906 (13)	0.0567 (10)	0.1022 (14)	−0.0145 (9)	−0.0602 (11)	0.0188 (9)
N3	0.0500 (9)	0.0432 (8)	0.0505 (9)	−0.0191 (7)	−0.0202 (7)	0.0050 (7)
C3	0.0358 (9)	0.0496 (10)	0.0399 (9)	−0.0125 (8)	−0.0105 (7)	0.0008 (7)
S4	0.0666 (3)	0.0398 (3)	0.0594 (3)	−0.0221 (2)	−0.0330 (3)	0.0094 (2)
O4	0.0534 (8)	0.0396 (7)	0.0538 (8)	−0.0150 (6)	−0.0225 (6)	0.0052 (6)
N4	0.0575 (10)	0.0443 (9)	0.0500 (9)	−0.0191 (7)	−0.0244 (7)	0.0048 (7)
C4	0.0408 (10)	0.0469 (10)	0.0387 (9)	−0.0130 (8)	−0.0099 (7)	−0.0016 (7)
C5	0.0322 (9)	0.0530 (10)	0.0381 (9)	−0.0136 (7)	−0.0070 (7)	−0.0002 (8)
C6	0.0329 (9)	0.0512 (10)	0.0434 (9)	−0.0129 (7)	−0.0084 (7)	0.0039 (8)
C7	0.0424 (10)	0.0569 (12)	0.0515 (11)	−0.0151 (9)	−0.0098 (8)	0.0013 (9)
C8	0.0518 (12)	0.0536 (12)	0.0756 (15)	−0.0155 (10)	−0.0120 (11)	−0.0060 (10)
C9	0.0525 (13)	0.0535 (12)	0.0885 (17)	−0.0163 (10)	−0.0142 (11)	0.0128 (12)
C10	0.0649 (15)	0.0684 (14)	0.0657 (14)	−0.0224 (11)	−0.0186 (11)	0.0252 (12)
C11	0.0554 (12)	0.0638 (13)	0.0487 (11)	−0.0201 (10)	−0.0139 (9)	0.0073 (9)
C12	0.0551 (12)	0.0499 (11)	0.0563 (12)	−0.0133 (9)	−0.0248 (9)	0.0056 (9)
C13	0.0423 (10)	0.0378 (9)	0.0394 (9)	−0.0129 (7)	−0.0094 (7)	0.0012 (7)
C14	0.0419 (10)	0.0391 (9)	0.0399 (9)	−0.0125 (7)	−0.0115 (7)	0.0016 (7)
C15	0.0418 (10)	0.0377 (9)	0.0456 (10)	−0.0134 (7)	−0.0119 (8)	0.0033 (7)
C16	0.0411 (10)	0.0416 (9)	0.0380 (9)	−0.0139 (7)	−0.0081 (7)	0.0002 (7)
C17	0.0419 (10)	0.0496 (10)	0.0391 (9)	−0.0179 (8)	−0.0046 (7)	−0.0051 (7)
C18	0.0532 (12)	0.0622 (12)	0.0491 (11)	−0.0252 (10)	−0.0164 (9)	0.0030 (9)
C19	0.0531 (12)	0.0857 (16)	0.0502 (11)	−0.0313 (11)	−0.0171 (9)	−0.0032 (11)
C20	0.0600 (14)	0.0822 (16)	0.0566 (12)	−0.0385 (12)	−0.0090 (10)	−0.0175 (11)
C21	0.0719 (16)	0.0575 (13)	0.0790 (16)	−0.0310 (11)	−0.0153 (12)	−0.0107 (11)
C22	0.0540 (12)	0.0494 (11)	0.0671 (13)	−0.0195 (9)	−0.0170 (10)	−0.0051 (9)

### 5-(3-Phenylisothiazol-5-yl)-1,3,4-oxathiazol-2-one (I) Geometric parameters (Å, º)

**Table:**

S1—N1	1.6845 (17)	C7—C8	1.380 (3)
S1—C1	1.7616 (19)	C7—H7A	0.9300
O1—C1	1.185 (2)	C8—C9	1.380 (3)
N1—C2	1.271 (2)	C8—H8A	0.9300
C1—O2	1.391 (2)	C9—C10	1.376 (4)
S2—N2	1.6406 (18)	C9—H9A	0.9300
S2—C3	1.7071 (18)	C10—C11	1.382 (3)
O2—C2	1.364 (2)	C10—H10A	0.9300
N2—C5	1.327 (2)	C11—H11A	0.9300
C2—C3	1.449 (3)	C13—C14	1.447 (3)
S3—N3	1.6790 (16)	C14—C15	1.365 (3)
S3—C12	1.746 (2)	C15—C16	1.417 (3)
O3—C12	1.187 (3)	C15—H15A	0.9300
N3—C13	1.278 (2)	C16—C17	1.479 (3)
C3—C4	1.361 (3)	C17—C22	1.386 (3)
S4—N4	1.6457 (17)	C17—C18	1.387 (3)
S4—C14	1.7084 (18)	C18—C19	1.384 (3)
O4—C13	1.361 (2)	C18—H18A	0.9300
O4—C12	1.385 (2)	C19—C20	1.380 (4)
N4—C16	1.329 (2)	C19—H19A	0.9300
C4—C5	1.417 (3)	C20—C21	1.371 (4)
C4—H4B	0.9300	C20—H20A	0.9300
C5—C6	1.476 (3)	C21—C22	1.386 (3)
C6—C7	1.390 (3)	C21—H21A	0.9300
C6—C11	1.398 (3)	C22—H22A	0.9300
			
N1—S1—C1	93.13 (8)	C9—C10—H10A	119.6
C2—N1—S1	109.43 (14)	C11—C10—H10A	119.6
O1—C1—O2	122.21 (17)	C10—C11—C6	120.3 (2)
O1—C1—S1	131.23 (16)	C10—C11—H11A	119.9
O2—C1—S1	106.56 (13)	C6—C11—H11A	119.9
N2—S2—C3	94.78 (9)	O3—C12—O4	122.3 (2)
C2—O2—C1	111.28 (14)	O3—C12—S3	130.15 (18)
C5—N2—S2	110.54 (13)	O4—C12—S3	107.58 (13)
N1—C2—O2	119.58 (17)	N3—C13—O4	120.27 (17)
N1—C2—C3	124.86 (17)	N3—C13—C14	123.90 (17)
O2—C2—C3	115.55 (16)	O4—C13—C14	115.82 (16)
N3—S3—C12	93.31 (9)	C15—C14—C13	128.92 (17)
C13—N3—S3	108.63 (13)	C15—C14—S4	109.41 (14)
C4—C3—C2	129.89 (17)	C13—C14—S4	121.66 (14)
C4—C3—S2	108.82 (14)	C14—C15—C16	110.62 (16)
C2—C3—S2	121.28 (14)	C14—C15—H15A	124.7
N4—S4—C14	94.50 (9)	C16—C15—H15A	124.7
C13—O4—C12	110.20 (15)	N4—C16—C15	115.12 (16)
C16—N4—S4	110.35 (13)	N4—C16—C17	119.36 (16)
C3—C4—C5	111.36 (16)	C15—C16—C17	125.51 (16)
C3—C4—H4B	124.3	C22—C17—C18	118.80 (18)
C5—C4—H4B	124.3	C22—C17—C16	120.93 (18)
N2—C5—C4	114.50 (17)	C18—C17—C16	120.26 (17)
N2—C5—C6	119.40 (16)	C19—C18—C17	120.2 (2)
C4—C5—C6	126.10 (16)	C19—C18—H18A	119.9
C7—C6—C11	118.40 (19)	C17—C18—H18A	119.9
C7—C6—C5	121.30 (17)	C20—C19—C18	120.6 (2)
C11—C6—C5	120.29 (18)	C20—C19—H19A	119.7
C8—C7—C6	120.7 (2)	C18—C19—H19A	119.7
C8—C7—H7A	119.7	C21—C20—C19	119.5 (2)
C6—C7—H7A	119.7	C21—C20—H20A	120.2
C7—C8—C9	120.5 (2)	C19—C20—H20A	120.2
C7—C8—H8A	119.7	C20—C21—C22	120.3 (2)
C9—C8—H8A	119.7	C20—C21—H21A	119.8
C10—C9—C8	119.4 (2)	C22—C21—H21A	119.8
C10—C9—H9A	120.3	C17—C22—C21	120.6 (2)
C8—C9—H9A	120.3	C17—C22—H22A	119.7
C9—C10—C11	120.7 (2)	C21—C22—H22A	119.7

### 5-(3-Phenylisothiazol-4-yl)-1,3,4-oxathiazol-2-one (II) Crystal data

**Table:**

C_11_H_6_N_2_O_2_S_2_	*F*(000) = 536
*M**_r_* = 262.30	*D*_x_ = 1.602 Mg m^−^^3^
Monoclinic, *P*2_1_/*c*	Mo *K*α radiation, λ = 0.71073 Å
*a* = 9.7202 (6) Å	Cell parameters from 6714 reflections
*b* = 9.9723 (6) Å	θ = 2.7–28.3°
*c* = 11.2165 (7) Å	µ = 0.48 mm^−^^1^
β = 90.399 (1)°	*T* = 296 K
*V* = 1087.22 (12) Å^3^	Block, colorless
*Z* = 4	0.48 × 0.43 × 0.37 mm

### 5-(3-Phenylisothiazol-4-yl)-1,3,4-oxathiazol-2-one (II) Data collection

**Table:**

Bruker APEXII CCD diffractometer	2228 reflections with *I* > 2σ(*I*)
φ and ω scans	*R*_int_ = 0.017
Absorption correction: multi-scan (SADABS; Bruker, 2008)	θ_max_ = 27.0°, θ_min_ = 2.9°
*T*_min_ = 0.719, *T*_max_ = 0.837	*h* = −12→12
8041 measured reflections	*k* = −12→9
2362 independent reflections	*l* = −14→14

### 5-(3-Phenylisothiazol-4-yl)-1,3,4-oxathiazol-2-one (II) Refinement

**Table:**

Refinement on *F*^2^	Hydrogen site location: inferred from neighbouring sites
Least-squares matrix: full	H-atom parameters constrained
*R*[*F*^2^ > 2σ(*F*^2^)] = 0.030	*w* = 1/[σ^2^(*F*_o_^2^) + (0.0465*P*)^2^ + 0.4061*P*] where *P* = (*F*_o_^2^ + 2*F*_c_^2^)/3
*wR*(*F*^2^) = 0.083	(Δ/σ)_max_ = 0.001
*S* = 1.02	Δρ_max_ = 0.37 e Å^−^^3^
2362 reflections	Δρ_min_ = −0.28 e Å^−^^3^
155 parameters	Extinction correction: SHELXL2014 (Sheldrick, 2015), Fc^*^=kFc[1+0.001xFc^2^λ^3^/sin(2θ)]^-1/4^
0 restraints	Extinction coefficient: 0.018 (2)

### 5-(3-Phenylisothiazol-4-yl)-1,3,4-oxathiazol-2-one (II) Special details

**Table:**

Geometry. All esds (except the esd in the dihedral angle between two l.s. planes) are estimated using the full covariance matrix. The cell esds are taken into account individually in the estimation of esds in distances, angles and torsion angles; correlations between esds in cell parameters are only used when they are defined by crystal symmetry. An approximate (isotropic) treatment of cell esds is used for estimating esds involving l.s. planes.

### 5-(3-Phenylisothiazol-4-yl)-1,3,4-oxathiazol-2-one (II) Fractional atomic coordinates and isotropic or equivalent isotropic displacement parameters (Å^2^)

**Table:**

	*x*	*y*	*z*	*U*_iso_*/*U*_eq_	
S2	0.45224 (4)	0.17168 (4)	0.50567 (3)	0.04262 (13)	
S1	0.88687 (4)	0.63218 (4)	0.59213 (4)	0.04650 (14)	
O1	0.65815 (9)	0.53305 (10)	0.65111 (8)	0.0330 (2)	
C1	0.74809 (14)	0.63165 (15)	0.69017 (12)	0.0365 (3)	
C3	0.62185 (13)	0.35992 (13)	0.50912 (11)	0.0288 (3)	
O2	0.72696 (12)	0.69783 (14)	0.77664 (11)	0.0542 (3)	
C2	0.70630 (13)	0.47087 (13)	0.55025 (10)	0.0288 (3)	
N2	0.55493 (14)	0.18187 (13)	0.38899 (11)	0.0427 (3)	
N1	0.82008 (12)	0.51010 (13)	0.50596 (10)	0.0395 (3)	
C4	0.51836 (13)	0.30589 (14)	0.57667 (12)	0.0330 (3)	
H4	0.4894	0.3384	0.6501	0.040*	
C5	0.63936 (13)	0.28477 (14)	0.40146 (11)	0.0325 (3)	
C6	0.74116 (14)	0.31005 (14)	0.30578 (11)	0.0337 (3)	
C11	0.83741 (15)	0.21201 (15)	0.27851 (13)	0.0386 (3)	
H11A	0.8384	0.1317	0.3206	0.046*	
C7	0.73957 (18)	0.42883 (17)	0.24153 (14)	0.0472 (4)	
H7A	0.6757	0.4951	0.2595	0.057*	
C10	0.93225 (15)	0.23383 (18)	0.18830 (14)	0.0465 (4)	
H10A	0.9976	0.1687	0.1710	0.056*	
C9	0.92952 (19)	0.3518 (2)	0.12467 (15)	0.0530 (4)	
H9A	0.9927	0.3662	0.0641	0.064*	
C8	0.8330 (2)	0.4488 (2)	0.15076 (15)	0.0560 (4)	
H8A	0.8307	0.5280	0.1070	0.067*	

### 5-(3-Phenylisothiazol-4-yl)-1,3,4-oxathiazol-2-one (II) Atomic displacement parameters (Å^2^)

**Table:**

	*U*^11^	*U*^22^	*U*^33^	*U*^12^	*U*^13^	*U*^23^
S2	0.0448 (2)	0.0372 (2)	0.0460 (2)	−0.01130 (15)	0.00795 (16)	−0.00051 (15)
S1	0.0414 (2)	0.0518 (3)	0.0465 (2)	−0.01591 (16)	0.01521 (16)	−0.01786 (17)
O1	0.0314 (4)	0.0366 (5)	0.0311 (4)	−0.0004 (4)	0.0070 (3)	−0.0061 (4)
C1	0.0343 (6)	0.0385 (7)	0.0368 (7)	0.0007 (5)	0.0045 (5)	−0.0079 (6)
C3	0.0290 (6)	0.0297 (6)	0.0277 (6)	0.0022 (5)	0.0018 (4)	0.0015 (5)
O2	0.0487 (6)	0.0632 (7)	0.0508 (6)	−0.0034 (5)	0.0123 (5)	−0.0286 (6)
C2	0.0309 (6)	0.0307 (6)	0.0248 (5)	0.0034 (5)	0.0032 (4)	−0.0011 (5)
N2	0.0485 (7)	0.0387 (7)	0.0409 (6)	−0.0091 (5)	0.0072 (5)	−0.0070 (5)
N1	0.0380 (6)	0.0447 (7)	0.0359 (6)	−0.0089 (5)	0.0103 (5)	−0.0122 (5)
C4	0.0341 (6)	0.0325 (6)	0.0325 (6)	0.0005 (5)	0.0038 (5)	0.0025 (5)
C5	0.0345 (6)	0.0321 (6)	0.0310 (6)	0.0003 (5)	0.0017 (5)	−0.0014 (5)
C6	0.0359 (7)	0.0375 (7)	0.0277 (6)	−0.0039 (5)	0.0018 (5)	−0.0069 (5)
C11	0.0390 (7)	0.0394 (7)	0.0375 (7)	−0.0018 (6)	−0.0013 (5)	−0.0091 (6)
C7	0.0556 (9)	0.0439 (9)	0.0422 (8)	0.0062 (7)	0.0132 (7)	0.0012 (7)
C10	0.0381 (7)	0.0568 (10)	0.0448 (8)	−0.0001 (7)	0.0056 (6)	−0.0179 (7)
C9	0.0525 (9)	0.0679 (11)	0.0389 (8)	−0.0095 (8)	0.0164 (7)	−0.0097 (8)
C8	0.0709 (11)	0.0543 (10)	0.0432 (8)	−0.0021 (8)	0.0166 (8)	0.0065 (7)

### 5-(3-Phenylisothiazol-4-yl)-1,3,4-oxathiazol-2-one (II) Geometric parameters (Å, º)

**Table:**

S2—N2	1.6546 (13)	C5—C6	1.4864 (18)
S2—C4	1.6827 (14)	C6—C7	1.386 (2)
S1—N1	1.6820 (12)	C6—C11	1.389 (2)
S1—C1	1.7463 (14)	C11—C10	1.391 (2)
O1—C2	1.3750 (14)	C11—H11A	0.9300
O1—C1	1.3849 (17)	C7—C8	1.383 (2)
C1—O2	1.1921 (17)	C7—H7A	0.9300
C3—C4	1.3737 (18)	C10—C9	1.376 (3)
C3—C5	1.4324 (17)	C10—H10A	0.9300
C3—C2	1.4511 (18)	C9—C8	1.380 (3)
C2—N1	1.2770 (17)	C9—H9A	0.9300
N2—C5	1.3208 (18)	C8—H8A	0.9300
C4—H4	0.9300		
			
N2—S2—C4	95.45 (6)	C3—C5—C6	127.12 (12)
N1—S1—C1	93.61 (6)	C7—C6—C11	119.48 (13)
C2—O1—C1	111.27 (10)	C7—C6—C5	121.01 (13)
O2—C1—O1	122.58 (13)	C11—C6—C5	119.50 (13)
O2—C1—S1	130.46 (12)	C6—C11—C10	120.07 (15)
O1—C1—S1	106.96 (9)	C6—C11—H11A	120.0
C4—C3—C5	110.58 (12)	C10—C11—H11A	120.0
C4—C3—C2	122.58 (12)	C8—C7—C6	120.03 (15)
C5—C3—C2	126.64 (11)	C8—C7—H7A	120.0
N1—C2—O1	118.86 (12)	C6—C7—H7A	120.0
N1—C2—C3	126.82 (12)	C9—C10—C11	120.04 (15)
O1—C2—C3	114.23 (11)	C9—C10—H10A	120.0
C5—N2—S2	109.98 (10)	C11—C10—H10A	120.0
C2—N1—S1	109.27 (9)	C10—C9—C8	119.98 (15)
C3—C4—S2	109.25 (10)	C10—C9—H9A	120.0
C3—C4—H4	125.4	C8—C9—H9A	120.0
S2—C4—H4	125.4	C9—C8—C7	120.40 (17)
N2—C5—C3	114.74 (12)	C9—C8—H8A	119.8
N2—C5—C6	118.14 (12)	C7—C8—H8A	119.8
